# Parental perspectives concerning gluten-free food insecurity in households with children with coeliac disease: take away messages for policy makers

**DOI:** 10.1017/jns.2026.10131

**Published:** 2026-07-30

**Authors:** Diana R. Mager, Sven Anders, Marcia Bruce, Justine M. Turner

**Affiliations:** 1 Department of Agricultural, Food and Nutritional Sciences, Faculty of Agricultural, Food & Environmental Science, University of Alberta, Canada; 2 Department of Resource Economics & Environmental Sociology, Faculty of Agricultural, Life and Environmental Science, University of Alberta, Canada; 3 Faculty of Medicine, University of Calgary, Canada; 4 Department of Pediatrics, Faculty of Medicine & Dentistry, University of Alberta, Canada

**Keywords:** Caregiver perspectives, Food insecurity, Gluten-free diet, Policy, social media

## Abstract

Coeliac disease (CD) is an autoimmune disease resulting in lifelong intolerance to gluten. Treatment of CD is a gluten-free diet (GFD). High gluten-free (GF) food costs contribute to GF-food insecurity (GF-FI) in households with children with CD. The study objective was to describe parental perceptions of the barriers/facilitators of GF-food security (GF-FS) and the health policies parents deem necessary to combat GF-FI. A subset analysis of a cross-sectional study examining household and child socio-demographic, child diet quality and GFD adherence, food environment, and GF-FI in parent–child dyads (*n* = 59) using validated questionnaires. Virtual interviews included 13 open-and-closed ended questions examining parent perceptions related to barriers/facilitators of GF-FS and health policies to address GF-FI. Questions were validated for face-and-content using an iterative process with parental responses deductively coded using the 5As of food insecurity theoretical framework. High GF-food costs (>90%) and the lack of effective GF-food specific health policies (∼94%) were the most commonly cited barriers to GF-FS, particularly in GF-FI households. Social media (>60%) was used to share information related to tax credits (15%) affordability, accessibility, and availability of GF-foods (75%). Parents deemed child tax credits (∼75%), development of food policies focused on education in schools (∼34%), dietary prescriptions (25%) and official recognition of CD as a disability (∼26%) as important to address GF-FI. In conclusion, parents perceive high GF-food costs and lack of effective GF-food health policies as important mediators of GF-FI. Social media platforms were widely used by the community to share information and solutions to address GF-FI.

## Introduction

Coeliac disease (CD) is an autoimmune disease resulting in gluten intolerance.^(1)^ Gluten is a protein found in barley, wheat, and rye. The main treatment for CD is lifelong adherence to a gluten-free diet (GFD).^(2)^ One of the greatest challenges for families with children with CD, is the high costs of GF-foods which can range between 150% and 200% higher than similar gluten containing foods.^(3)^ Previous work has shown that gluten-free food insecurity (GF-FI) can range between 30% and 50% in households with children with CD in Canada, with more than 2/3 of families experiencing moderate-to-severe GF-FI.^(4,5)^ The consequences of GF-FI in households with children with CD includes lower child adherence to the GFD and reduced diet quality.^(4–6)^ All of these factors place the child with CD living in GF-FI households at high risk for long-term health consequences such as poor bone health and/or growth failure.

In Canada, there are limited government policies to address the high rates of GF-FI for children and adults living with CD. The current taxation credit that is in place for Canadians living with CD is complex and cumbersome to use, requiring the calculation of marginal costs between GF-foods and comparable gluten containing food items.^(7)^ In addition, the tax credit doesn’t address the burden of high GF-food costs directly, and thus has no direct benefit to families without taxable incomes.^(7,8)^ While other government policy approaches have been proposed (e.g. defined child tax or disability credit), there are no specific health policies for families with children with CD to address GF-FI. Hence, parents living in GF-FI households are faced with the dilemma of making decisions regarding income allocation between high GF-food costs and other important household expenditures (e.g. rent/mortgage payments).

Little is known regarding parental perceptions related to the mediators of GF-FI in households with children with CD or the health policies that parents deem to be important. The study aim was to describe parental perceptions regarding perceived barriers and facilitators of household GF-FS and to explore the food policies parents deem necessary to help combat household GF-FI.

## Methods

This is an analysis of the qualitative data from a multi-method, cross-sectional study that included a quantitative arm that was conducted in parents and youth (2–18 years) with CD and a qualitative arm that examined parents’ perspectives (qualitative data) about needed policy formation to address gluten-free food insecurity (GF-FI). The quantitative arm of the study examined GF-FI, sociodemographic characteristics (child, parent, and household), the food environment, child diet quality, and parental literacy regarding the GFD.^(5)^ Participants (children and their parents or responsible caregivers) were recruited (Feb 2024–March 2025) using recruitment fliers disseminated in the Pediatric Celiac Clinic at the Stollery Children’s Hospital, social media platforms (e.g., Instagram and Facebook) run by Celiac Canada (local, national chapters) and celiac community groups (e.g., community CD-focused groups) as described previously.^(5)^ Households with children with CD living outside of Canada, or with children <2 years or not on the GFD, and children with multiple food allergies or additional medical diagnoses necessitating additional dietary alterations, such as in Type 1 Diabetes, were excluded.

Two interviews were conducted virtually on Zoom® by trained research assistants. The first interview was done to collect quantitative information related to household, parent and child socio-demographic and anthropometric characteristics, child clinical information related to CD diagnosis, and assessment of household GF-FI using validated food insecurity surveys (2-item Hunger Vital, Six-Item Short Form Food Insecurity Modules) as described previously.^(5,9,10)^ GF-FI severity was determined according to the six-item HFSSM according to raw scores of i) zero (high GF-food security or high FS), ii) 1–4.9 (marginal GF-FS), iii) 1.5–4.49 (low GF-FS and iv) 4.6–6 (very low GF-FS) as described previously.^(4,10,11)^ Diagnosis with CD in the children was confirmed according to standard clinical guidelines.^(5,12,13)^ Socio-demographic information collected from the first interviews regarding child (age, age at CD diagnosis, weight-*z,* and height-*z*), parents (age, education, and ethnicity) and household (income, urban/rural residency, number of parents/responsible caregivers) were included in this analysis.^(5,14,15)^

A second virtual interview (qualitative data) was conducted with parents to determine parental perceptions regarding the mediators of household GF-FI, and the food policies parents deemed necessary to combat or address GF-FI. All interviews were conducted by a primary trained research assistant (RA), with an additional trained research assistant who taped interviews and took ‘field notes’ during each interview. The interviews were independently transcribed verbatim by two additional graduate trainees and were cross verified by the primary research assistant who conducted all of the interviews to ensure accuracy in transcription. Thematic analysis was then independently completed by the primary research assistants conducting the interviews and the RA responsible for taking field notes/taping the interviews. All themes were then cross verified to ensure consistency. Where there was a lack of consensus, the PI made the determining decision after review.

Parental perspectives were evaluated from the responses to both open- and close-ended questions that focused on parent identified barriers/facilitators of household GF-FS (*n* = 9 questions) and what support/food policies parents indicated were needed (*n* = 3 questions) to address GF-FI (Supplementary Table 1). Content for these questions were based upon responses to an open-ended question by parents in a recent Canadian cross-country survey examining the prevalence of GF-FI in Canadian households with children with CD.^(4)^ This study elicited important information about parental reported mediators of GF-FI (affordability, accessibility, agency, availability, and adequacy) and provided important content and context for interview questions developed in this study.^(4)^ Interview question content and format were also validated for content and face validity by interdisciplinary health care professionals (RD, MD, RN; *n* = 6) in the field by an iterative process.

Research ethics approval was obtained from the Human Research Ethics Board (Pro00126345) at the University prior to study initiation. Participant informed consent and assent were obtained prior to study enrollment.

## Data analysis

Socio-demographic, anthropometric, and clinical data related to child CD diagnosis was expressed as mean ± SD or median and interquartile range (IQR) for parametric and non-parametric data, respectively. Shapiro–Wilk test was conducted to assess the normality of distribution. Levene’s test was conducted to assess the homogeneity of variances. Independent *t*-tests and Mann Whitney tests were used to determine differences between groups for parametric and non-parametric data, respectively. Categorical data were analysed using chi-square and/or fisher exact test. These analyses were performed using Statistical Analysis Software (SAS, Version 9.4; SAS Institute Inc., Cary, NC). A *p* value ≤ 0.05 was considered statistically significant.

Parental responses to open-ended and close-ended questions were analysed by manifest content analysis.^(16)^ Thematic codes were developed deductively using the 5A’s framework (e.g. availability, accessibility, adequacy, acceptability, and agency) as a starting point by trained investigators. When new themes were identified (e.g. affordability), then an additional category was created.^(17)^ Thematic coding was performed independently by two trained study personnel and then cross checked for accuracy and auditing by a third trained individual. When consensus of data coding was not achieved, the principal investigator made the determining decision. Agreement for theme identification by members of the research team were analysed using intraclass correlation coefficients (ICC) classifications (poor-fair agreement [ICC < 0.4], fair-moderate agreement [0.41–0.60], good agreement [0.61–0.8], and excellent agreement [0.81–1]).^(18)^ Agreement for theme identification was excellent (>0.90). Data were reviewed until no new recurring themes were identified, indicating thematic saturation.

## Results

### Child, parental and household socio-demographics

A total of 59 parents representing 54 parent-1 child and *n* = 5 parent-2 child groupings were recruited as described previously, participated in the qualitative arm of the study.^(5)^ Children were diagnosed with CD between 5–11 years of age, had age-appropriate rates of growth since CD diagnosis, with no differences between GF-FS and GF-FI households. CD diagnosis was confirmed by small bowel biopsy (∼41%) and by serological diagnosis (∼59%) according to standard guidelines.^(12,13)^ The majority of parents (maternal/paternal) were Caucasian (>80 %), aged 42–45 years (>90%), had university/CGEP or college education (>80%), were living in two parent-households (>90%) with no differences between GF-FI and GF-FS households (*p* > 0.05). Most GF-FS households were two-parent households (>93%), located in urban centres (>58%), with household incomes (>$100 K CAD) well above the poverty level.^(19)^ In contrast, GF-FI households were more often located in rural areas (GF-FI: 5/12 vs GF-FS:16/47 *p* = 0.04) with household incomes <$100K CAD (GF-FI: 6/12 vs GF-FS: 4/47 *p* < 0.001) than GF-FS households.

### Barriers of GF-FS: GF-food acceptability, accessibility, affordability, adequacy, and agency

Representative quotes are presented in Table 1. Parents reported the lack of appropriate government support (agency) related to ineffective tax credits (55/59), particularly in GF-FI households (*p* = 0.02), as the major barrier to household GF-FS. This finding remained significant when adjusted for home ownership, rural vs urban residency, household income or dual/single parent households. Fewer parents living in GF-FI households felt they had adequate supports (agency) in the community (GF-FI: 2/12 vs GF-FS: 21/47; *p* = 0.001) to purchase affordable GF-foods (GF-FI: 5/12 vs 39/47 *p* = 0.01) that met their child’s nutritional needs (GF-FI: 1/12 vs GF-FS: 42/47; *p* = 0.001) when compared to parents in GF-FS households (adequacy and affordability). When adjusted for the potential confounding impact of household income and urban/rural residency, these findings only remained significantly different when urban/rural residency was included in the model with more parents living in GF-FI rural households being concerned about the lack of supports in the community in relation to the affordability of GF-foods. One important representative quote included: ‘*difficult for people living outside big cities to get sufficient and healthy gluten free foods if they heavily rely on local small stores that do not cater to specialized diets (and special conditions: snow, bad road conditions*)’.

**Table 1.** Thematic analysis of parental perceptions of barriers and facilitators of GF-FS and food policies Table 1 long description.

Themes	Representative quotes from parents of children with celiac disease
**Barriers to gluten-free food security**	
Affordability^a^	“*High food cost of food and gluten-free foods.”* “*Gluten-free foods are really expensive. Some companies have stopped making gluten-free food that they* *relied on before.”* *“Does it truly cost that much more to make that item or you are taking advantage by selecting a population that* *has no choice but to eat this way.”*
Availability^b^	“*Global economic, trade agreements, Canada is so isolated and relies on imports a lot. I worry it will become more* *challenging for companies to import into Canada”.* “*Options for gluten-free foods are decreasing since those companies do not want to do business in Canada* *anymore.”*
Adequacy^c^	“*Yes, but concerned about nutritional content; GF products are very sugary and fat, nutrient poor, lack of fiber* *and protein. Child would rather not eat; she would rather go hungry. Its really expensive”.* “*GF-foods: generally lack of fibre is a big issue, so they have to make up for it by having more fruits and* *vegetables etc. supplementing it by taking daily gummy fiber…”* “*Fresh GF-bread product is not always good quality (dry and flaky, moldy inside) and it can be very expensive”.*
Accessibility^d^	“*Difficult for people to get sufficient and healthy foods (and/or gluten-free foods) if they heavily rely on* *local small stores that do not cater to specialized diets (and special conditions: snow, bad road conditions).”* “*Accessibility (grocery not nearby) school cafeteria (limited options; availability of gluten-free foods) nothing* *her children could get except apples. Should give a certain level of consideration to the school and* *organizational level who interface with those kids.”*
Agency: lack of government support (high GF-food costs)^e^	“*Little help for parents.”* “*None of those regulators have children with CD. If they did (government), they would ask for a better* *tax system”.*
Inability to access knowledgeable Health care providers on a routine basis.^f^	“*We need to see a registered dietitian or a knowledgeable family doctor made available for the patient.* *Can be really hard to get access.”*
**Facilitators of gluten-free food security^g^**	
Social media use	“*Local facebook group in an individual provincial Celiac Chapter (very active) provide information* *about what GF-foods are on sale”.* “*An app called “Find me GF” help to find gluten-free restaurants.”* “*Local Celiac Canada Chapter Facebook group specific to CD; useful resources that are helpful to* *find GF restaurants/specific GF brands”*
Community support: family, School, health care providers	“*Parents and friend support; family support”* “*Gluten free support group in Alberta (i.e. a support group) they can come together to learn how to read label,* *what kind of gluten-free food are there in their nearby town”*
**Recommended gluten free-food policies for Canadians with CD^h^**	
Tax credit	“*Cost of gluten-free; part of celiac group tax break, complicated and hard to claim; need support at this level”.* *Celiac disease is becoming more present in the younger population, they don’t have extra incomes, and this could* *also put a strain on healthcare if they get sick.”* *“Tax credit system is like a part-time job”*
Education policy (schools, health providers, regulatory agencies)	“*Work with teachers and children to know about celiac disease; important for people to understand* *it’s not a choice. If we eat gluten, our children get sick period.”*
Official disability recognition	“*Make GF food more affordable; tax benefit, “disability” credit, financial support for young people with celiac* *disease; discounted food; medical coverage”.*
Diet prescriptions for GFD	“*In the UK (the participant’s mom was diagnosed in the UK), they can get prescriptions for their basic gluten-free* *foods. As for low-income families in the UK, the prescription is free (guarantee those people with low income can* *get gluten-free foods). They don’t do it now so much, but it was amazing.”*
Food subsidies (cap on GF-foods)	*“People who are diagnosed with CD could be given either a stipend or a relevant law saying that gluten-free foods* *are at the same price as gluten equivalent so that everybody has access to affordable gluten-free foods.”*

The following definition was employed (https://www.torontomu.ca/foodsecurity/ unless specified).

a Affordability: The ability to afford food at all times (77.8% or 44/56) had issues with affordability.

b Availability: Sufficient supply of food for all people at all times. (39% or 22/56) had issues with availability.

c Adequacy: Access to food that is nutritious and safe, and produced in an environmentally sustainable way. More parents concerned with adequacy of GF-foods in GF-FI (6/7 or 85.7%) vs GF-FS (8/41 or 19.5 %); *p* = 0.002.

d Accessibility: Physical and social access to food for all people at all times. 12.5% of households (7/56) reported difficulties with accessibility of GF-Foods.

e Agency: The policies and processes that enable the achievement of food security**.** 94% (53/56 households) reported lack of effective government policies.

f Limited Health professional Access (RD, MD): 77.8% (44/56) indicated that this was a problem; 3/56 had no access (5.4%); no difference between GF-FI and GF-FS.

g Facilitators of GF-FS: i) Social Media: A total of 28 parents responded; *N* =17/28 (60.7%) indicated that social media was important facilitator to address GF-FI.

h Food Policy: Tax credit (*n* = 42/56 or 75%), Education or School Policy (*n* = 19/56 or 33.9%), Disability Recognition for CD (*n* = 15/56 or 26.8%), Diet Prescriptions for Gluten-free diet (*n* = 14/56 or 25%), Food Subsidies (retailer caps on GF-foods: 9/56 or 16%).

CD, celiac disease; GFD, gluten-free diet; GF, gluten free; GF-FI, gluten-free food insecurity; GF-FS, gluten-free food security.

Additional barriers to GF-FS identified included the lack of availability of GF-foods (39%) and the inability to routinely access nutrition specialists with expertise related to the GFD (77.8%). These findings were consistent in both GF-FI and GF-FS households (*p* > 0.05). A greater number of parents (GF-FI: 40/47 vs GF-FS: 2/12; *p* = 0.002) living in GF-FI households were more worried that the nutritional quality and safety of the GF-foods they purchased for their child compared to parents living in GF-FS households (Table 1).

### Facilitators of household GF-FS

Representative quotes are presented in Table 1. The major facilitators of household GF-FS identified by parents included the importance of CD focused social media (60.7%; *n* = 17/28) with no differences noted between GF-FI and GF-FS households (*p* > 0.05) observed. Social media was used to i) share information about policy initiatives related to tax credits (15% or 8/56) by Celiac Canada (national/chapters) and other community groups ii) affordability, accessibility, and availability of GF-foods in local regions (43/56), iii) education related to CD (14/56), and iv) potential donations of GF-foods to local food banks/pantries (35% or 20/56). While additional supports were highlighted (e.g. local food banks, community kitchens, health professionals) as potential facilitators of GF-FS, less than 10% of the parents (GF-FS and GF-FI households *p* > 0.05) reporting any recent use of these options. A representative quote from the parents’ perspective included ‘*Local Celiac Canada social media groups that are specific to CD are the most helpful. They provide useful resources that are helpful to find GF restaurants/specific GF brands’.*

### Health policies and GF-FS

Over 75% (42/56) of parents indicated that making the tax system easier via institution of a more effective tax credit (e.g. refundable tax credit) was important to address GF-FI. An important illustrative parent quote that addressed this issue included ‘*tax credit system is not helpful at all, is waste of time and the government makes us do all the work and we still get denied’*. Other policies highlighted were the need for dietary prescriptions for GF-foods (14/56) GF-food subsidies or caps on GF-food pricing (9/56) identification of CD as an official disability (15/56) by regulators, and development of food policies that would provide education to health providers, government agencies, and educators in the schools (19/56). These findings were consistent between GF-FI and GF-FS households (*p* > 0.05) and across reported household incomes, geographical residency (urban vs rural) or home ownership (*p* > 0.05). Additional representative quotes are presented in Table 1.

### Barriers and facilitators of adherence to the GFD in GF-FI and GF-FS households

Self-reported child adherence to the GFD was >90% in this cohort. No differences in parental identified barriers (food costs, social or school settings, selective child eating patterns, travel) or facilitators (home GF-food preparation, support in community settings by parents, family and educators, age of the child) of GFD adherence were noted between GF-FI and GF-FS households (*p* > 0.05). The most important determinant as reported by parents of child adherence to the GFD identified was the presence of parent/family and educator support (44/56) in the community with no differences reported between GF-FI and GF-FS households (*p* > 0.05). An important representative illustrative quote included: ‘*Experience a whole life without gluten. very cautious and committed, read ingredients, talking to everyone in my child’s life. Family support (e.g. labelling food products in the fridge), his teachers and principal about lunch programs (have some gluten-free food products e.g. granola bar), but you can’t always be sure that the food isn’t contaminated with gluten’.*

## Discussion

This analysis examined parents’ perceptions about the mediators contributing to GF-FI and the health policies that are needed to address GF-FI in households with children with CD. High GF-food costs and the lack of effective government policies to address high GF-food costs were the main factors parents’ highlighted as contributing factors to GF-FI. Other factors such as the lack of accessible and adequate GF-foods and health professionals in the community to help families combat GF-FI were also highlighted. Parents utilised a variety of approaches to address these issues including the predominant use of CD-specific social media accounts (e.g., Facebook, Instagram) run by national or regional/provincial chapters of Celiac Canada or other community groups. These local social media groups provided critical information related to cost-effective or less expensive sources of GF-foods at restaurants, local grocery or specialty GF-food food outlets. Uniformly, parents of children with CD, indicated that a refundable child tax credit would be the most useful government policy to combat high GF-food prices. Other recommendations highlighted the potential for regulation aimed at ‘capping’ GF-food prices, the consideration of medically derived GF-food prescriptions, identification of a CD diagnosis as a disability and increased education to school, health providers and government agencies about the criticality of implementing effective GF-food policies by regulators. The latter was also highlighted as an important strategy to ensure adherence to the GFD by children and youth with CD, particularly in school settings.

Study findings regarding parent’s perceptions of high GF-food costs as an important mediator of GF-FI are consistent with the literature. High GF-food costs have been well documented, particularly in relation to food staples such as GF-breads, pasta, and cereals where food prices are often more than 200% the cost of their gluten containing counterparts.^(3,20)^ This is likely to be an ongoing challenge for families with children with CD. Global food inflation continues to rise since the onset of COVID-19 with the most recent 2025 statistics indicating that global food inflation has risen by 3–5%.^(21,22)^ This includes naturally occurring GF-foods such as fruits and vegetables. Much of this ongoing rise in food prices has been associated with climate change, global conflicts, tariffs and fluctuating markets; all have contributed to increasing rates of FI globally.^(11,23)^ The most recent Canadian statistics relating to FI indicate that over 10 million Canadians (25–30%) experience FI; including 2.5 million children.^(24)^ This is even more alarming in households with children with CD; whereby GF-FI has been shown to occur in 30–50% of households.^(4,6)^ This occurs even in households with incomes well above the poverty line. These differences also exist in northern and Indigenous communities, where grocery prices are typically much higher; exacerbated by high food transportation costs and lack of competitive grocery food purchasing options with many residents having access to one grocery store.^(25,26)^ Hence study findings confirm the need to urgently develop effective food policies in Canadian families with children with CD living with GF-FI.

Recently, Canada released a new National School Food Policy Program^(27)^ to address the high rates of food insecurity in Canada, but significant gaps in terms of funding and implementation have been identified.^(28)^ Moreover, this program does not include the dietary needs of children with therapeutic dietary requirements such as the GFD, where lifelong adherence to the GFD is critical to prevent long term adverse health sequelae and reduced health related quality of life. A recent systematic review from 12 countries from Europe, Asia, South America, Australia, and Africa has shown that the introduction of food policies related to offsetting high GF-food costs (e.g. food allowances, deductible tax credits) and improving health care provider support in adults with CD was associated with some improvements in subdomains of health related quality of life (HRQOL: emotional, social, worry) that are known to be important to GFD adherence.^(29)^ These findings are consistent with study findings indicating the need to also develop food policies that focus on both offsetting high GF-food costs (e.g. deductible tax credits) and education provided to health care professionals, schools, and regulatory agencies as these policies will address GFD adherence and GF-FI.

Development of other food policies related to GF-diet prescriptions have met with variable success. While these policies have been implemented in a variety of countries, dietary prescriptions for GF-foods are not available in Canada. Factors such as lack of awareness or limitations in the range of GF-food products provided and withdrawal of these polices by some countries due to financial constraints have all raised concerns about the longevity and effectiveness of these policy approaches.^(30–32)^ Food allowances or applying GF-food capping economics are additional approaches that could be considered to address high GF-food costs for Canadians, but require concerted financial supports by regulators. Moreover, even in the US, many federal nutrition programs such as the Supplemental Nutrition Assistance Program, National School Lunch Programs and Special Supplemental Nutrition for Woman, Infants, and Children have had funding reduced and do not explicitly address GF-foods. These findings all highlight the importance of establishing sustainable and effective food policies for families with children with CD.

Some factors may have influenced study findings. The use of social media as a primary modality for participant recruitment may have resulted in a recruitment bias towards parents who frequently use social media; thereby resulting in the emergence of social media as the important platform to exchange information related to CD and policy formation reported in this study. Use of various social media platforms (e.g. Facebook, Twitter, TikTok, and Instagram) in relation to sharing of information related to CD diagnosis, food and nutrition literacy, availability, accessibility and affordability of GF-foods has been well documented within the literature by adults with CD including parents of children with CD, particularly in the post COVID-19 era.^(33–38)^ While this may have informed parental responses in relation to the importance and use of social media to discuss proposed mediators of GF-FI and potential health care policies such as refundable child tax credits, this has also been a point of discussion within the Canadian Celiac community over the past 10 years whereby the current system to receive tax credits has been deemed difficult and not beneficial to lower income Canadians with CD.^(7)^ Use of social media may have also introduced a potential recruitment bias to more digitally competent populations versus clinic-based participants who may have also differed in their health perceptions.^(39, 40)^ However, no differences in socio-demographic characteristics (parent and child) or parent perceptions related to mediators of GF-FI or necessary health care policies were found between participants recruited via social media or in clinic.

Social media literacy, type, and usage may also be impacted by the presence of food insecurity and lower household socio-economic status whereby households with food insecurity may have more negative outlooks on information related to health and disease.^(41)^ This may have influenced parent’s perspectives about both the mediators contributing to GF-FI and potential government policies they deemed necessary. While parents predominantly lived in urban, higher income households and had post-secondary education levels, there were no major differences in the major themes associated with identification of mediators of GF-FI (high GF-food costs, ineffective government health policies) between GF-FI and GF-FS households. While it is possible that a more ethnically diverse population of parents, with varying socio-economic status, may have identified differences in ‘ranking of interventions/government policies’ to address high GF-food costs and current government approaches, this study did not show any of these differences. In fact, uniformly parents in both GF-FS and GF-FI households ranked a change in the taxation system (refundable child tax credit) as the number one priority to address the high costs of GF-foods. Consideration of more immediate sources of help (food allowances, diet prescriptions, food caps) may be particularly important for families with household incomes below the poverty line and those living in northern areas of Canada where food insecurity is over 50%.^(25)^ These types of food policies may also help to address many of the concerns parents have related to lack of GF-food availability and affordability. Ongoing work in this avenue of investigation is warranted.

## Conclusions

Parent’s perceive high GF-food costs and ineffective government policies related to the current tax credits for children with CD and specific food policies as major barriers to GF-FS in their households. Use of community social media platforms run by provincial and national chapters of Celiac Canada and other community groups were the main strategies employed by families to address high GF-food costs and their concerns related to reliable access to healthy and nutritious GF-foods. Parental recommendation for refundable tax credits for children with CD, along with official recognition of CD as a disability and/or dietary prescriptions were viewed as important strategies to address the high rates of GF-FI experienced by this community. This information should be used to guide future health policy to address FI in households with children with CD.

## Supporting information

10.1017/jns.2026.10131.sm001 Mager et al. supplementary material Mager et al. supplementary material

## Supplementary material

The supplementary material for this article can be found at https://doi.org/10.1017/jns.2026.10131.

## Table 1. Long description

The table is divided into several sections: Barriers to gluten-free food security, Facilitators of gluten-free food security, and Recommended gluten-free food policies for Canadians with CD. Each section contains themes and representative quotes from parents. The Barriers section includes themes such as Affordability, Availability, Adequacy, Accessibility, and lack of government support. The Facilitators section includes themes like Social media use and Community support. The Recommended policies section includes themes like Tax credit, Education policy, Official disability recognition, Diet prescriptions for GFD, and Food subsidies. Each theme is accompanied by quotes from parents that illustrate the theme. The table has multiple rows and columns, with each row representing a different theme and each column providing representative quotes.

Navigate back to Table 1..
