# Supplementary material for: Parental perspectives concerning gluten-free food insecurity in households with children with coeliac disease: take away messages for policy makers
Source: J Nutr Sci. 2026 Jul 30;15:e69. doi: 10.1017/jns.2026.10131 (PMC13439013; doi:10.1017/jns.2026.10131)
Supplement: Supplementary file 1 [file S2048679026101311sup001.docx]

**Supplementary Table 1:** Open Ended Questions for Assessment of Parental Perspectives related to Barriers and Facilitators of Gluten-Free Food Insecurity (GF-FI) and Strategies/Interventions

| Question 1 (Availability) | Do you think sufficient and diverse food is always available in your community? How about gluten-free foods specifically? |
| --- | --- |
| Question 2 (Accessibility) | Are you able to easily access adequate and acceptable foods? How about gluten-free foods specifically? |
| Question 3 (Affordability) | Are you able to afford adequate and acceptable foods? How about gluten-free foods specifically? |
| Question 4 (Adequacy) | Are the foods of good quality (safe and nutritional)? Are gluten-free foods you purchased good quality? |
| Question 5 (Acceptability) | Are you able to access some traditional foods (gluten-free foods) that reflect the types of foods that are culturally appropriate (which can also bring you enjoyment from consuming the food)? |
| Question 6 (Agency) | Are there any local supports that are helpful for you to address the high costs of foods and/or accessibility of foods? |
| Question 7 (Barriers to Food Security) | What do you think are the barriers to food security for your children and/or your household? |
| Question 8 (Facilitators/Food Security) | What do you think are the facilitators of food security for your children and/or your household? |
| Question 9 (Facilitators of Adherence to the gluten-free diet with GF-FI) | What do you think are the facilitators of your child's adherence to the GFD? |
| Question 10 (Strategies to Address GF-FI) | What are some potential intervention strategies that you think would be helpful for your family in terms of food security? |
| Question 11 (Ranking of Most Useful Strategies parents employ) | If you have to choose one intervention strategy that you use (as above) which one would you choose? |
| Question 12 (Other) | Is there anything else you would like to share with us? |
